# Advances in the Genetics of Hypertension: The Effect of Rare Variants

**DOI:** 10.3390/ijms19030688

**Published:** 2018-02-28

**Authors:** Alessia Russo, Cornelia Di Gaetano, Giovanni Cugliari, Giuseppe Matullo

**Affiliations:** 1 Department of Medical Sciences, University of Turin, 10126 Turin, Italy; alessia.russo@hugef.org (A.R.); cornelia.digaetano@unito.it (C.D.G.); giovanni.cugliari@hugef.org (G.C.); 2 Italian Institute for Genomic Medicine (IIGM, Formerly HuGeF), 10126 Turin, Italy

**Keywords:** essential hypertension, blood pressure, genome-wide association studies, exome microarray, next-generation sequencing, rare variants, rare-variants association testing, burden test, sequence kernel association test

## Abstract

Worldwide, hypertension still represents a serious health burden with nine million people dying as a consequence of hypertension-related complications. Essential hypertension is a complex trait supported by multifactorial genetic inheritance together with environmental factors. The heritability of blood pressure (BP) is estimated to be 30–50%. A great effort was made to find genetic variants affecting BP levels through Genome-Wide Association Studies (GWAS). This approach relies on the “common disease–common variant” hypothesis and led to the identification of multiple genetic variants which explain, in aggregate, only 2–3% of the genetic variance of hypertension. Part of the missing genetic information could be caused by variants too rare to be detected by GWAS. The use of exome chips and Next-Generation Sequencing facilitated the discovery of causative variants. Here, we report the advances in the detection of novel rare variants, genes, and/or pathways through the most promising approaches, and the recent statistical tests that have emerged to handle rare variants. We also discuss the need to further support rare novel variants with replication studies within larger consortia and with deeper functional studies to better understand how new genes might improve patient care and the stratification of the response to antihypertensive treatments.

## 1. Introduction

Systemic hypertension is a consistently elevated systolic or diastolic blood pressure in the systemic arteries. Systolic blood pressure (SBP) is generated by the contraction of the ventricles and represents the highest blood pressure (BP) level. Diastolic blood pressure (DBP) is the BP remaining during the relaxation of the ventricles and represents the lowest BP level. The term Pulse Pressure (PP) refers to the difference (in mmHg) between the systolic and diastolic pressures, while the Mean Arterial Pressure (MAP) is the average BP during a single cardiac cycle [1,2,3]. Clinicians consider 140 mmHg as the maximum normal adult SBP value, and 90 mmHg as the upper limit for normal DBP value, as suggested by the World Health Organization (WHO) [4]. Usually, high SBP is caused by the narrowing of the arterioles. This narrowing raises the peripheral resistance to blood flow, which requires a greater workload for the heart and raises arterial pressure [1]. Elevated BP levels still represent a huge public health issue worldwide, being the major risk factor for cardiovascular disease, including coronary heart disease, stroke, and heart failure. Each year, 17 million people prematurely die because of cardiovascular disease, and, among these, nine million deaths occur as a consequence of hypertension-related complications [5].

Ninety-five percent of hypertensive patients presents a type lacking an obvious identifiable cause (Essential or Primary Hypertension). Investigations of twin and family studies revealed a moderate heritability ranging between 30% and 50% [6,7]. Hypertension is a heterogeneous disease; besides genetic variation, several factors such as age, sex, and ethnicity influence this trait, in addition to other environmental factors (e.g., lipid levels and obesity).

So far, the study of hypertension has mostly been based on Genome-Wide Association Studies (GWAS). GWAS represent a valuable approach to type hundreds of thousands of Single Nucleotide Polymorphisms (SNPs) in very large cohorts. During the last ten years, many studies have been published thanks to the setting of very large consortia, including the International Consortium for Blood Pressure Genome-Wide Association Studies, Cohorts for Heart and Aging Research in Genomic Epidemiology (CHARGE) and Global BPgen, the Wellcome Trust Case Control Consortium Studies, the UK Biobank, the PBCHARGE-EchoGen consortium, the CHARGE-HF consortium [8,9,10,11,12], leading to the identification of more than 100 SNPs implicated in BP levels, as recently reviewed by Seidel and Scholl [13].

The cause of a complex trait, like essential hypertension, remains elusive if examined in the light of the GWAS results. There has been a step forward compared to the classic GWAS analyses thanks to system genetics approaches and related statistical methods [14]. These approaches use intermediate phenotypes, such as transcript, protein, or metabolite levels, and quantify and integrate them with several traits of interest. Several genes pathways and networks underlying common human diseases have been discovered using systems genetics studies.

For example, data derived from GWAS were integrated with expression data to provide a measure of functional variation, i.e., the expression Quantitative Trait Loci (eQTL). When one of these loci is located within ≤1 Mb from the gene encoding the transcript, it is termed a *cis*-eQTL. When an eQTL affects the expression level of a distal gene, it is called *trans*-eQTL. Disease susceptibility can be regulated by a plethora of genes controlled by *trans*-eQTLs which, for this reason, are very informative [15].

Thanks to studies based on rat, mouse, or human cells and tissues, it has been calculated that about 30% of mammalian genes are under the control of eQTLs and they heavily contribute to complex disease susceptibility [16]. Moreover, using comparative genomics between established rat models of hypertension and humans, several studies have shown that human genes found to be associated with hypertension through GWAS, when conserved in the rat, are likely to form both *cis*- and *trans*-acting eQTLs in multiple tissues [17]. These studies have also taken advantage of a statistical methods known as Weighted Gene Co-Expression Network Analysis (WGCNA) that studies biological networks based on pairwise correlations between variables and is often used to highlight clusters (modules) of highly correlated genes [18].

At the heart of the GWAS-based approaches lies the “common variant–common disease” hypothesis. However, when considering all the detected high-frequency variants in aggregate, the percentage of BP variability explained by genetic variants accounts for only 2–3%. Moreover, blood pressure changes related to different genotypes at these loci are estimated to be modest, approximately 1.0 and 0.5 mmHg for SBP and DBP, respectively [19]. Considering the moderate effects and the scarce genetic control ascribable to high-frequency variants, two possible scenarios came forward: a wrong heritability was estimated, or alleles are more likely to be heterogeneous and uncommon. Furthermore, array-based technologies were infrequently conducted for the detection of causal polymorphisms. These observations implicated strong limits in exploiting GWAS to identify druggable targets with high confidence and supported the idea that rare (frequency < 1%) and uncommon (frequency between 1% and 5%) functional variants may explain a greater fraction of hypertensive individuals. The arrival of Next-Generation Sequencing (NGS) technologies facilitated a shift in focus from common to rare variants and provided the opportunity to unravel the genomic architecture underlying hypertension risk. Along with the development of even more advanced laboratory methodologies, statistical genetic models must also evolve to meet the challenge of using rare variants to link previously unidentified genome loci to BP changes [20,21].

In this review, we first present an overview of the most recent findings regarding the role of rare and uncommon variants in BP alteration identified through the currently available technologies, moving from the candidate-gene approach to the high-throughput exome chips, and then to NGS solutions; next, we report the statistical methods proposed so far for rare variants analysis. Finally, we draw conclusions on the contribution ascribable to rare and low-frequency variants in the improvement of cardiovascular risk assessment.

## 2. Results

### 2.1. Results from Studies on Selected Single Nucleotide Variants and Genes

Conducting studies based on a candidate-gene approach is the easiest and cheapest way to investigate genetic variation. *FBN1*, a gene that is thought to be causative of vascular damage and whose mutations have been previously detected only in relation to Marfan syndrome, was selected by Jeppesen and colleagues [22] as their research focus. A sample of 4839 Danish subjects was genotyped for the rs11856553 rare variant (Minor Allele Frequency, MAF, of A allele = 0.2%, 1000 Genomes) using a PCR-based method. In the Health 2006 study, an unadjusted risk of hypertension of 2.67 (95% Confidence Interval, CI, 1.14–6.18) for the G/A genotype was reported. The adjusted risk of moderate to severe hypertension (grade 3) for the A/A–G/A genotypes (homozygous and heterozygous carriers were grouped) was 8.01 (95% CI, 3.27–19.58), *p* < 0.0001). No significant differences in BP between G/A and G/G variant carriers were described within the MONICA10 study, however, the adjusted risk of moderate to severe hypertension (grade 2) for A/A-G/A variants was 6.54 (2.12–20.2); *p* < 0.01. It is still undefined how this intronic mutation could functionally affect hypertension [22].

The cytokine Interleukin-6 (IL-6) is a fundamental mediator of the acute-phase response to endothelial injury and regulates the production of C Reactive Protein (CRP) in hepatocytes [23]; therefore, both *IL-6* and *CRP* genetic variants have been evaluated in relation to hypertension [2,20]. In the paper from Karaman et al. [24], *IL-6* rs1800795 and rs1800796 SNPs (MAF = 14.12% and 31.39%, respectively, 1000 Genomes) were genotyped in a Turkish sample of 108 controls and 111 hypertension patients. Both SNPs genotypes were not significantly related to hypertension or to IL-6 and CRP plasma levels. The CC genotype of the rs1800796 SNP is very rare in the examined population and large frequency differences among different populations and geographic regions have been reported [25].

Endothelial nitric oxide synthase (eNOS) produces Nitric Oxide, a vasodilator of vascular smooth muscles, and thus plays a crucial role in regulating BP. A four-SNP haplotype, comprising the uncommon variant rs11699009 in the *BPIFB4* gene, has been associated with notable longevity [26]. In the study of Vecchione et al. [27], 416 individuals were genotyped to determine their haplotypes. The rare variant-haplotype carriers showed a significantly increased DBP (*p* = 0.013) and a borderline increased SBP (*p* = 0.067). The authors demonstrated that the overexpression of the *BPIFB4* uncommon variant in mice impaired eNOS signaling and increased BP, opening the way for the development of new therapeutic strategies.

### 2.2 Results from Exome Chips-Based Studies

When the 1000 Genomes Project became publicly available, data from NGS technology allowed the development, from Affymetrix (Santa Clara, CA, USA) and Illumina (San Diego, CA, USA) companies, of array-based genotyping platforms which offer the possibility to capture a greater range of single nucleotide variability compared to GWAS. In Table 1, studies investigating common and rare variants in association with hypertension andBP phenotypes and through exome array approaches are listed. Most publications [28,29,30,31,32,33,34,35,36,37,38]; (Table 1) took advantage of the Illumina HumanExome BeadChip (Exome Chip; Illumina, Inc., San Diego, CA, USA).

This chip was produced in order to meet the need of moving from relatively frequent variants derived from GWAS to functional variants located in coding regions. The array constitutes an intermediate choice between GWAS and NGS of large number of samples in terms of both cost and practical issues. The Exome Chip was designed on genome and exome sequencing data of 16 contributing studies, reaching a total of 12,031 subjects. In the array, 247,039 markers were assayed including 84% rare variants, 9.2% low-frequency variants, and only 5.8% common variants which were identified more than three times in at least two different datasets. Most variants (>90%) are non-synonymous or splicing variants that were absent in previously available chips. Genotyped individuals were mostly of European American ancestry which led to some concerns about the evolutionary young age of variants and population-specific results. The Exome Chip consortia provided information on several common diseases, including cardiovascular disease (Available online: http://genome.sph.umich.edu/wiki/Exome_Chip_Design) [39].

In 2015, Sung and colleagues [28] reported the results of rare and low-frequency single variants and four sets of gene-based analyses using Exome Chip data on 2045 African-American subjects from the HyperGEN cohort. Neither Single Nucleotide Variants (SNVs) nor gene level analyses reached genome-wide Bonferroni-corrected thresholds (*p* < 6.4 × 10^−7^ for SNVs; *p* < 2 × 10^−6^ with MAF < 1% and *p* < 3.9 × 10^−6^ with MAF < 5%) [28].

The same cohort was used for analyses focused on both rare (through the Exome Chip) and common (using the AffymetrixGenome-WideHumanSNP6.0 Array) variants within the *PCSK9* gene in relation to BP traits. PCSK9 is a protease able to interact with the three subunits of the renal epithelial sodium channel (ENaC). This interaction consequently increases proteasomal degradation of the ENaC which regulates sodium reabsorption [40]. Among the 31 SNPs identified, none of the associations were statistically significant (*p* > 0.05). The cumulative effect of rare variants (mostly non-synonymous or stop-gain SNVs) detected in *PCSK9* was significantly associated with DBP in HyperGEN (*p* = 0.04) and to SBP in REGARDS data (*p* = 0.04). The disparity in the associated phenotypes was probably due to differences in the age of populations [29].

Alteration in lipid levels is strongly related to hypertension [41], and a pleiotropic effect of lipid-associated loci on hypertension could be speculated. To investigate this, the group of Kim et al. [38] interrogated 135 Exome Chip SNVs for associations with ten cardiometabolic traits in 14,028 Korean individuals. Three new common variants in the *BRAP*, *ACAD10*, and *ALDH2* genes within the 12q24.12 locus were significantly associated with both SBP and DBP (*p* < 1.09 × 10^−4^; effect sizes between −1.53 ± 0.32 and −0.78 ± 0.20). The locus was also associated with High-Density Lipoprotein (HDL), Low-Density Lipoprotein (LDL), triglycerides, fasting plasma glucose, body mass index, and waist–hip ratio (*p* < 1.06 × 10^−2^; effect sizes between −7.60 ± 1.72 and 2.55 ± 0.53) [38]. Successively, a longitudinal Exome-Wide Association Study (EWAS), which is a genotyping method restricted to exonic SNVs using Illumina exome chips, allowed the detection of six hypertension-related SNVs at the 12q24.1 locus, creating an East Asian-specific haplotype comprising five derived alleles. The study was conducted in 6026 Japanese individuals whose disease progression and physiological changes were traced for several years during annual health check-ups. The rationale of this study was the observation that SBP, DBP, and the prevalence of hypertension are significantly correlated with age, while conventional GWAS have commonly been conducted in a cross-sectional manner measuring traits at a single point in time. People carrying the East Asian-specific haplotype displayed a hypertension prevalence significantly lower than those individuals carrying a common haplotype (mean Odds Ratio (OR) = 0.78, *p* < 1.0 × 10^−8^). Furthermore, using a recessive model, an SNV located within the *COL6A5* gene, was significantly associated with SBP (Estimate: −2.93; *p* = 2.3 × 10^−8^) [30].

Stop codons are highly likely to alter protein function affecting BP-related traits, and, for this reason, Ohlsson et al. [31] focused the aim of their work on the relationship between BP and protein truncating variants in the genotypes of 5453 Swedish people. They reported 19 SNVs associated with SBP with a *p* value < 0.05. The *PDE11A* R307X mutation conferred a 7 mmHg higher SBP and a 4.6 mmHg higher DBP (β coefficients = 7.0 (1.8–12) for SBP corrected, *p* = 0.009 and 4.6 (1.8–7.4) for DBP corrected, *p* = 0.001) and was previously described as a loss-of-function mutation linked to familial hypertension and Cushing’s syndrome [42]. The stop codon mutation caused a three-fold increased risk of hypertension in female carriers (OR = 3.1 (95% CI, 1.3–7.4), *p* = 0.009).

Two very large meta-analyses were then published at the same time to identify novel coding variants and loci influencing BP traits and hypertension. In the first meta-analysis, Surendran et al. [32] genotyped 192,763 subjects, mostly of European descent, in the discovery phase. Fifty-one genomic regions were found to be significantly associated with at least one of the following BP traits: SBP, DBP, PP, and hypertension in the discovery analysis (*p* < 5 × 10^−8^). Thirty novel SNVs replicated in 155,063 multiethnic populations (*p* < 6.2 × 10^−4^; βs: −1.43−2.70). Among these, rare putative functional variants were identified within *A2ML1*, *COL21A1*, *RRAS*, *RBM47*, and *ENPEP* genes. Interestingly, intersecting previous GWAS data with Exome Chip data revealed five out 35 known loci which likely had rare coding functional variants. The second large meta-analysis was conducted on all ancestry subjects from the same five consortia described in Surendran et al. [32], reaching a total sample number of 327,288. Here, the authors identified 31 additional new loci with statistically significant associations with one of the BP traits (*p* < 3.4 × 10^−7^). Three variants had frequencies between 1% and 5% and were non-synonymous substitutions in *NPR1* (already established), *SVEP1*, and *PTPMT1* (novel genes) with a *p* value less than 3.4 × 10^−7^ when corrected for multiple testing. To note, the BP increment attributable to any of these low-frequency variants (>1.5 mmHg) was higher than any of the novel common SNPs described here. Low-frequency and frequent SNVs with non-synonymous, stop-coding, and splicing effects were aggregated using burden tests to identify new gene-based associations. These analyses showed significant results for *NPR1* (*p* = 4.4 × 10^−5^) and marginally for *PTPMT1* and *DBH* genes (*p* = 0.019 and 0.053, respectively). Considering that an overlap between cardiovascular-specific pathways and metabolic disease-related factors was observed, the authors suggested a shared origin between the phenotypes that could be exploited for new drugs discovery [33].

Two very large meta-analyses were then published at the same time to identify novel coding variants and loci influencing BP traits and hypertension. In the first meta-analysis, Surendran et al. [32] genotyped 192,763 subjects, mostly of European descent, in the discovery phase. Fifty-one genomic regions were found to be significantly associated with at least one of the following BP traits: SBP, DBP, PP, and hypertension in the discovery analysis (*p* < 5 × 10^−8^). Thirty novel SNVs replicated in 155,063 multiethnic populations (*p* < 6.2 × 10^−4^; βs: −1.43−2.70). Among these, rare putative functional variants were identified within *A2ML1*, *COL21A1*, *RRAS*, *RBM47*, and *ENPEP* genes. Interestingly, intersecting previous GWAS data with Exome Chip data revealed five out 35 known loci which likely had rare coding functional variants. The second large meta-analysis was conducted on all ancestry subjects from the same five consortia described in Surendran et al. [32], reaching a total sample number of 327,288. Here, the authors identified 31 additional new loci with statistically significant associations with one of the BP traits (*p* < 3.4 × 10^−7^). Three variants had frequencies between 1% and 5% and were non-synonymous substitutions in *NPR1* (already established), *SVEP1*, and *PTPMT1* (novel genes) with a *p* value less than 3.4 × 10^−7^ when corrected for multiple testing. To note, the BP increment attributable to any of these low-frequency variants (>1.5 mmHg) was higher than any of the novel common SNPs described here. Low-frequency and frequent SNVs with non-synonymous, stop-coding, and splicing effects were aggregated using burden tests to identify new gene-based associations. These analyses showed significant results for *NPR1* (*p* = 4.4 × 10^−5^) and marginally for *PTPMT1* and *DBH* genes (*p* = 0.019 and 0.053, respectively). Considering that an overlap between cardiovascular-specific pathways and metabolic disease-related factors was observed, the authors suggested a shared origin between the phenotypes that could be exploited for new drugs discovery [33].

Two very large meta-analyses were then published at the same time to identify novel coding variants and loci influencing BP traits and hypertension. In the first meta-analysis, Surendran et al. [32] genotyped 192,763 subjects, mostly of European descent, in the discovery phase. Fifty-one genomic regions were found to be significantly associated with at least one of the following BP traits: SBP, DBP, PP, and hypertension in the discovery analysis (*p* < 5 × 10^−8^). Thirty novel SNVs replicated in 155,063 multiethnic populations (*p* < 6.2 × 10^−4^; βs: −1.43−2.70). Among these, rare putative functional variants were identified within *A2ML1*, *COL21A1*, *RRAS*, *RBM47*, and *ENPEP* genes. Interestingly, intersecting previous GWAS data with Exome Chip data revealed five out 35 known loci which likely had rare coding functional variants. The second large meta-analysis was conducted on all ancestry subjects from the same five consortia described in Surendran et al. [32], reaching a total sample number of 327,288. Here, the authors identified 31 additional new loci with statistically significant associations with one of the BP traits (*p* < 3.4 × 10^−7^). Three variants had frequencies between 1% and 5% and were non-synonymous substitutions in *NPR1* (already established), *SVEP1*, and *PTPMT1* (novel genes) with a *p* value less than 3.4 × 10^−7^ when corrected for multiple testing. To note, the BP increment attributable to any of these low-frequency variants (>1.5 mmHg) was higher than any of the novel common SNPs described here. Low-frequency and frequent SNVs with non-synonymous, stop-coding, and splicing effects were aggregated using burden tests to identify new gene-based associations. These analyses showed significant results for *NPR1* (*p* = 4.4 × 10^−5^) and marginally for *PTPMT1* and *DBH* genes (*p* = 0.019 and 0.053, respectively). Considering that an overlap between cardiovascular-specific pathways and metabolic disease-related factors was observed, the authors suggested a shared origin between the phenotypes that could be exploited for new drugs discovery [33].

Two very large meta-analyses were then published at the same time to identify novel coding variants and loci influencing BP traits and hypertension. In the first meta-analysis, Surendran et al. [32] genotyped 192,763 subjects, mostly of European descent, in the discovery phase. Fifty-one genomic regions were found to be significantly associated with at least one of the following BP traits: SBP, DBP, PP, and hypertension in the discovery analysis (*p* < 5 × 10^−8^). Thirty novel SNVs replicated in 155,063 multiethnic populations (*p* < 6.2 × 10^−4^; βs: −1.43−2.70). Among these, rare putative functional variants were identified within *A2ML1*, *COL21A1*, *RRAS*, *RBM47*, and *ENPEP* genes. Interestingly, intersecting previous GWAS data with Exome Chip data revealed five out 35 known loci which likely had rare coding functional variants. The second large meta-analysis was conducted on all ancestry subjects from the same five consortia described in Surendran et al. [32], reaching a total sample number of 327,288. Here, the authors identified 31 additional new loci with statistically significant associations with one of the BP traits (*p* < 3.4 × 10^−7^). Three variants had frequencies between 1% and 5% and were non-synonymous substitutions in *NPR1* (already established), *SVEP1*, and *PTPMT1* (novel genes) with a *p* value less than 3.4 × 10^−7^ when corrected for multiple testing. To note, the BP increment attributable to any of these low-frequency variants (>1.5 mmHg) was higher than any of the novel common SNPs described here. Low-frequency and frequent SNVs with non-synonymous, stop-coding, and splicing effects were aggregated using burden tests to identify new gene-based associations. These analyses showed significant results for *NPR1* (*p* = 4.4 × 10^−5^) and marginally for *PTPMT1* and *DBH* genes (*p* = 0.019 and 0.053, respectively). Considering that an overlap between cardiovascular-specific pathways and metabolic disease-related factors was observed, the authors suggested a shared origin between the phenotypes that could be exploited for new drugs discovery [33].

Two very large meta-analyses were then published at the same time to identify novel coding variants and loci influencing BP traits and hypertension. In the first meta-analysis, Surendran et al. [32] genotyped 192,763 subjects, mostly of European descent, in the discovery phase. Fifty-one genomic regions were found to be significantly associated with at least one of the following BP traits: SBP, DBP, PP, and hypertension in the discovery analysis (*p* < 5 × 10^−8^). Thirty novel SNVs replicated in 155,063 multiethnic populations (*p* < 6.2 × 10^−4^; βs: −1.43−2.70). Among these, rare putative functional variants were identified within *A2ML1*, *COL21A1*, *RRAS*, *RBM47*, and *ENPEP* genes. Interestingly, intersecting previous GWAS data with Exome Chip data revealed five out 35 known loci which likely had rare coding functional variants. The second large meta-analysis was conducted on all ancestry subjects from the same five consortia described in Surendran et al. [32], reaching a total sample number of 327,288. Here, the authors identified 31 additional new loci with statistically significant associations with one of the BP traits (*p* < 3.4 × 10^−7^). Three variants had frequencies between 1% and 5% and were non-synonymous substitutions in *NPR1* (already established), *SVEP1*, and *PTPMT1* (novel genes) with a *p* value less than 3.4 × 10^−7^ when corrected for multiple testing. To note, the BP increment attributable to any of these low-frequency variants (>1.5 mmHg) was higher than any of the novel common SNPs described here. Low-frequency and frequent SNVs with non-synonymous, stop-coding, and splicing effects were aggregated using burden tests to identify new gene-based associations. These analyses showed significant results for *NPR1* (*p* = 4.4 × 10^−5^) and marginally for *PTPMT1* and *DBH* genes (*p* = 0.019 and 0.053, respectively). Considering that an overlap between cardiovascular-specific pathways and metabolic disease-related factors was observed, the authors suggested a shared origin between the phenotypes that could be exploited for new drugs discovery [33].

In the most recent meta-analysis on Exome Chip data, Nandakumar et al. [34] screened 15,914 individuals of African ancestry to detect novel genes and BP-related SNVs considering the full spectrum frequency. Nine rare SNVs (mostly missense) within eight genes (*SLC28A3*, *KRBA1*, *SEL1L3*, *YOD1*, *COL6A1*, *CRYBA2*, *GAPDHS*, and *AFF1*) exhibited Bonferroni-corrected associations with SBP or DBP (SeqMeta βs (βs_sm_): 21.10 (4.12)–73.65 (13.19); *p* < 4.6 × 10^−7^) and the *CCDC13, QSOX1* genes were also described through burden test including only predicted damaging variants (Betas_sm_: 54.38 (10.68) and 32.93 (7.13), respectively; *p* < 3.86 × 10^−6^). By contrast, no significant results were obtained considering common and low-frequency variations.

Linkage analysis can have good power to detect multiple rare or lower frequency BP variants in a gene or region with relatively larger effect sizes [43]. However, the identified linkage regions from well-designed linkage family studies [44,45,46] did not overlap with many BP loci identified by large BP GWAS of mostly unrelated individuals. Therefore, He and colleagues [35] applied variance-component linkage analysis to the Cleveland Family Study (CFS) to identify candidate genomic regions related to SBP, DBP, and PP. Since the region identified (16p13) showed no overlapping with any SNPs derived from previous GWAS, 517 individuals from the CFS who had been genotyped using the Illumina OmniExpress Exome array [39], were screened for variants within the 16p13 locus. At a gene-based level, the association between the aggregation of five rare variants within the *RBFOX1* gene and SBP as well as PP traits replicated in the meta-analysis of a large sample of 57,234 participants (*p* < 1.71 × 10^−2^). This gene encodes for the Ataxin-2 Binding Protein 1 whose genetic variations were suggested to have a protective effect on BP levels, although the underlying mechanisms remain to be clarified [35].

The UK Biobank is a huge prospective cohort including 500,000 individuals of European ancestry recruited to investigate genetic and non-genetic factors underlying diseases that takes advantage of many phenotypes and biological samples [47]. Genotypes obtained through a customized array in addition to genome-wide imputation based on 1000 Genomes and UK10K sequence data, and information related to BP traits, were retrieved for 140,886 participants included in the discovery phase of the study conducted by Warren and colleagues [37]. Both GWAS and exome analyses were performed to identify SNVs with MAF ≥ 1% and MAF ≥ 0.01%. Among the 240 loci derived from the discovery phase, 102 GWAS and five exome variants with *p* < 5 × 10^−8^ were reported. Noteworthy, a 9.3 mmHg higher SBP was observed after comparing subjects with the highest genetic risk score (estimated on the basis of all the loci identified) and above 50 years old with those with the lowest genetic risk score (95% CI: 6.9–11.7, *p* = 1 × 10^−13^) [37]. In the recently published paper from Pazoki and coauthors [48], the 267 SNPs identified by Warren et al. [37] were combined with the 47 BP-associated loci reported by Hoffman et al. [11] to calculate a genetic risk score for high BP in 277,005 subjects belonging to the prospective UK Biobank cohort. A healthy lifestyle score was also constructed for all the individuals in order to investigate whether the adherence to a favorable lifestyle could counteract the high genetic susceptibility to develop hypertension and cardiovascular diseases. The authors reported an association between healthy lifestyle and lower SBP and DBP within each tertile of genetic risk. In particular, at low genetic risk, the estimated mean SBP was 140 mmHg (95% CI, 102–177) among subjects with an unhealthy lifestyle and 134 mmHg (95% CI, 95–172) among those with a healthy lifestyle.

To date, the largest meta-analysis on exome chips data was conducted on 475,000 individuals (mostly European) genotyped using the UK BiLEVE array and the UK Biobank Axiom Array. These arrays are closely related new next-generation microarrays (95% identical content) designed from the Affymetrix Company. More than 800,000 markers were included to comprehensively cover beyond common SNPs, rare and low-frequency coding variants, copy number variants, pharmacogenomics markers, Human Leukocyte Antigen (HLA), inflammation, and eQTL variants. Among rare variants, in addition to primarily missense mutations, protein truncating variants resulting in premature stop codons, frameshifts, and loss of start mutations were included as loss-of-function variants. The genomic coverage was optimized for European and British populations. The array provided the opportunity to test the association between a wide range of genetic variations and many frequent human diseases, including cardiovascular disease and cardiometabolic traits such as BP (Available online: http://www.ukbiobank.ac.uk); [49]. In the paper from Kraja and colleagues [36], 21 SNVs showed significant associations with at least one BP trait, after correcting for multiple testing (*p* < 5 × 10^−8^; βs(se): −1.14 (0.19)–0.42 (0.06)). Moreover, all variants had concordant directions across all the datasets. Only one SNV (in the *DBH* gene) had a MAF less than 1% and exhibited the lowest effect estimate (βs(se): −1.14 (0.19); *p* = 1.23 × 10^−9^). Four novel associations of common SNPs within *SLC4A1AP*, *AFAP1, STAB1*, and *SYNPO2L* genes were reported [36].

### 2.3. Results from DNA Sequencing Studies

Large-scale genotyping through high-throughput platforms opened the way to great efforts aimed at discovering the causative variants explaining the associations described.

#### 2.3.1. Pre-Next-Generation Sequencing Era

Direct sequencing represented an easy way to characterize hypertension-related genes embedding SNPs found through GWAS. Okuda et al. [50] validated 143 SNPs identified in a small Japanese population. Among these SNPs, most had frequencies higher than 5% and caused amino acid substitutions, whereas almost all novel variants were rare (13 out of 16).

Genetic Epidemiology Network of Salt Sensitivity (GenSalt) study participants were recruited to evaluate SBP, DBP, and MAP responses to a dietary sodium intervention. The renin-angiotensin-aldosterone system (RAAS) is a hormonal cascade essential for the control of homeostasis, BP, and vascular tone [51,52]. In the first re-sequencing study focused on the RAAS pathway, Kelly and coauthors [53] analysed seven genes for putative associations with BP salt-sensitivity among participants of the GenSalt study. Carriers of 124 rare variants had 1.55-fold increased odds (95% CI: 1.15, 2.10) of salt sensitivity compared to non-carriers (*p* = 0.004). No genes showed significant associations with salt sensitivity after Bonferroni correction. No significant common and low-frequency single markers were detected when the analyses were corrected for multiple comparisons [53]. The reabsorption of sodium in epithelial cells located in the renal tubule is carried out by the renal epithelial sodium channel (ENaC) whose activity is fundamental for BP control [54]. *SCNN1A*, *SCNN1B*, and *SCNN1G* genes encode the three ENaC subunits [55]. These genes were targeted by Gu et al. [56] to identify novel common, low-frequency, and rare variants in 300 GenSalt participants with the highest MAP response to the high-sodium intervention and 300 GenSalt participants with the lowest MAP response to the high-sodium intervention. No significant associations with salt sensitivity were observed. In gene-based analyses, *SCNN1A* gene showed a significant association with salt sensitivity (*p* = 0.009). Individuals carrying rare variants in *SCNN1A* gene had an odds ratio of 0.52 (95% CI: 0.32–0.85). Neither *SCNN1G* nor *SCNN1B* associated with salt sensitivity in rare variant analyses. Three common variants in *SCNN1A* associated with salt sensitivity of BP (*p* < 1.3 × 10^−3^; 1.23-fold increased odds and 0.68–0.69-fold decreased odds of salt sensitivity) [56].

Another suggested candidate gene for hypertension is represented by the Cadherin-13 gene (*CDH13*). *CDH13* encodes a cell adhesion molecule involved in the protection of vascular endothelial cells from apoptosis following oxidative stress, survival, proliferation, and endothelial cells migration [57,58,59]. The promoter region was re-sequenced and subjected to methylation QTL (meQTL) analysis within the HYPertension in ESTonia (HYPEST) and Coronary Artery Disease in Czech (CADCZ) studies. The meQTL rs8060301 (a frequent variant) showed a pleiotropic effect on HDL and DBP (nominal *p* < 0.005), which was unconfirmed after multiple testing correction [60].

#### 2.3.2. Results from Next-Generation Sequencing Studies

GWAS identified more than 100 genetic variants influencing BP [13]. However, the causal variants underlying the majority of genetic associations remained unknown. In recent years, three different NGS approaches have been proposed to study rare variants in hypertension and BP (Table 2).

The first approach is to check GWAS signals and describe novel associations by performing a re-sequencing of only a few genes previously indicated by GWAS. This approach, commonly called target re-sequencing, is cheaper and allows one to highlight the variations within the whole frequency spectrum in a precise genomic locus. The strategy was adopted by the CHARGE Consortium. In the frame of this consortium, the signals identified by precedent GWAS were re-sequenced with the aim of describing novel variations with large effects on several common diseases [74]. Concerning BP, within the CHARGE Targeted Sequencing Study, target re-sequencing of 4178 Europeans was performed on six BP genes identified by GWAS (*ATP2B1*, *CACNB2*, *CYP17A1*, *JAG1*, *PLEKHA7*, and *SH2B3*), however, neither common nor rare variants were consistently associated with the trait with large effect sizes, independently of the original GWAS signals [63].

Regarding hypertension, an association with rs3918226 in the *eNOS* gene promoter was described in the GWAS from Salvi et al. [75] (OR for minor allele T = 1.34 (95% CI, 1.25–1.44); *p* = 1.03 × 10^−14^). In 2013, a 140 kb genomic area encompassing the *eNOS* gene was re-sequenced from the same group. The study identified 338 variants, including 61 novel variants, and rs3918226 still appeared as the SNP most closely associated with hypertension. Moreover, if compared with the C major allele, the T risk allele was associated with lower *eNOS* transcriptional activity when tested in HeLa cells [64].

A second approach is whole exome sequencing (WES) in which only the coding portions of the genome, (about 2%), estimated to harbor 85% of disease-causing mutations, are sequenced [76]. A WES study was performed on DNA samples from 17,956 individuals of European and African ancestries, included in the CHARGE, National Heart, Lung, and Blood Institute GO Exome Sequencing Project, Rotterdam Study, and the Erasmus Rucphen Family cohorts. These findings implicated the effect of the aggregation of 95 rare coding variants in *CLCN6* on decreasing BP levels of 3–4 mmHg, independently of the tagging SNP rs17367504 previously reported. The effect size described here was about four- to six-fold larger than previous common BP variants from GWAS [66].

Two additional studies exploited WES data to focus on selected genes. Loss-of-function mutations in *SLC12A3*, *SLC12A1*, and *KCNJ1* genes, essential for normal renal NaCl reabsorption, cause Bartter’s and Gitelman’s syndromes. Their exons were screened to search for rare heterozygous variants within the Framingham Heart Study offspring cohort. Thirty different mutations were observed. The mean long-term SBP among mutation carriers was 6.3 mmHg lower than the mean of the cohort (*p* = 0.0009). For DBP, the mean effect was −3.4 mmHg (*p* = 0.003) [62]. Findings from previous GWAS indicated *ULK4* and *MAP4* genes, encoding, respectively, a Serine/Threonine-Protein Kinase and a non-neuronal microtubule-associated protein, as related to BP and hypertension [8,77]. Thirty-six rare haplotype blocks were found to be significantly associated with BP in *ULK4* gene, and ten in *MAP4* gene [61]. The study described above was conducted in the frame of the Genetic Analysis Workshops (GAWs). Since 1982, GAWs were held by a group of multidisciplinary scientists to deal with the role of genetics in complex diseases. For GAW18, GT2D-GENES Consortium and the San Antonio Family Heart Study provided data on the whole genome, systolic and diastolic BP, and related covariates in two Mexican American samples. In the GAW19, new data were included reaching a collection of WGS, WES, and gene expression data from 20 large families in addition to a set of 1943 unrelated subjects whose exome sequences were available. Simulated phenotypes were also included for each sample on the basis of the real sequence data [78]. Several papers have been published so far, mostly on methodological approaches (see the following paragraph “Statistical analysis of rare variants”) to handle rare variations in relation to hypertension.

The third and most comprehensive NGS approach to examine the effect of rare variants is represented by WGS. Until now, to the best of our knowledge, only studies published within the GAWs analysed WGS data (Table 2) to search for genetic variations associated to hypertension, likely because a very large sample is needed to highlight rare variants, and this feature heavily affects the costs of the study. Three studies failed to identify significant associations after correction for multiple testing [68,69,72]. In the frame of the GAW18, Zhao et al. used novel sliding window approaches and a simulated dataset to analyse 142 unrelated individuals focusing on chromosome 3. The most significant windows fell into the known *MAP4* gene, considering both SBP and DBP. Other windows were reported within *SUMF* and *ARHGF3* genes in relation to DBP, and in *FLNB* and *BTD* for SBP [67]. Wang and Wei performed a gene-based genome-wide scan of 103 unrelated individuals to search for hypertension-associated genes. After using three different methods, only the *SETX* gene exhibited significant association. This gene consists of large intronic regions; indeed, most of the rare variants detected fall in intronic regions. The risk of hypertension, estimated after collapsing all the intronic variants, was 9.5 (OR = 9.5, 95% CI (3.43, 28.70); *p* = 8.8 × 10^−7^) [65]. Other significant findings were reported within the *MACROD1/LRP16* locus [70], *ADCY5*, and *UBE2E2* genes [71], and in an additional 23 genes [73] using different statistical approaches.

## 3. Statistical Analysis of Rare Variants

Gene-based association tests evaluate the relationship of rare variants enrichment in genes and phenotype or Mendelian and common diseases [79]. Region-based analysis has become the standard approach for analyzing rare variants, since standard individual variant tests are underpowered to detect rare variant effects because of the low allele frequencies. Statistical methods to test for rare variants can be categorized as burden approach [80,81,82] and SKAT (Sequence Kernel Association Test) approach [74,83]. Burden tests assume all rare variants in the target region have effects on the phenotype in the same direction and of similar magnitude [84,85], but they undergo a considerable loss of power in the presence of a large number of non-causal variants or in the presence of protective, deleterious, and null variants [86,87]. SKAT aggregates genetic information across the region using a kernel function and uses a computationally efficient variance component test to test for association. CMC (Combined Multivariate and Collapsing Method) collapses variants in subgroups according to allele frequencies and combines these subgroups using a T1 test [66,88]. Compared with population-based methods, family-based methods have more power and can prevent bias induced by population substructure [89]. The optimal weight was first proposed by Sha and coauthors in 2012 [90] in a population-based test called TOW (Test for the effect of an Optimally Weighted combination of variants) by assuming the independence among rare variants. FamSKAT [91], which accounts for familial correlation based on kinship coefficients in a linear mixed model, may be able to use both family and unrelated samples (developed for quantitative traits). Wang and coauthors in 2016 [92] proposed four weighting schemes for the family-based rare variants test (FBAT-v) [93]. Lee and coauthors in 2012 [94] derived the optimal test SKAT-O by estimating the correlation parameter in the kernel matrix to maximize the power, which corresponds to the estimated weight in the linear combination of the burden test and SKAT test statistics that maximizes power. Lin and coauthors in 2016 [68] extended the CAPL (Combined Association in the Presence of Linkage) [95] test, using both case-control and family data for testing from common variants to rare variant associations. A similarity-based weighted U approach is used to model the joint association analysis of sequencing variants and gene expression [69]. Sun and coauthors in 2016 [70] introduced a W-test collapsing method to evaluate rare variant associations by measuring the distributional differences between cases and controls through combined log of odds ratio within a genomic region. Wang and coauthors in 2016 [96] developed SKAT+, an estimation method that uses only control subjects; it has superior power over SKAT, while maintaining control over the type I error rate. Lu and coauthors in 2014 [71] reported the development and application of Trio-SVM (Support Vector Machine) approach that aggregates and evaluates the transmission of rare variants. The focus of Derkach and coauthors in 2014 [72] confirmed that Fisher’s method is not only robust but can also improve power over individual pooled linear and quadratic tests and is often better than other robust tests such as SKAT-O. Cao and coauthors in 2014 [73] developed a USR (Unified Sparse Regression) to incorporate prior information and jointly adjust for cryptic relatedness, population structure, and other environmental covariates; qMSAT (Quality-based Multivariate Score Association Test) [97] and SSU (Sum of Squared U) statistic tests [98] were equivalent to the SKAT.

## 4. Conclusions and Perspectives

Thanks to the introduction of exome arrays technologies, great efforts have been conducted to extend association analyses to rare and coding variants. Recently, the joint work of large consortia allowed the interrogation of hundreds of thousands of SNVs in up to 475,000 individuals [28,29,30,31,32,33,34,35,36,37,38]; (Table 1). Some new low-frequency and rare variants have been reported that are consistently associated with BP traits, with size effects higher than 1.5 mmHg, and that should undergo deep functional testing. Considering the single variant analyses described here, the largest effect, to date, was observed for a rare missense SNV in the *KLH3* gene in relation to SBP (8.2 mmHg with se = 4.1) [37]. Despite the large sample size (up to 422,604 subjects for the exome analysis), the study from Warren and coauthors was still underpowered to identify rare variants with statistical significance. When considering the joint impact of 107 mostly common variants, a 9.3 mmHg higher SBP was reported for subjects >50 years and carrying the highest genetic risk score [37]. This finding has potential implications concerning early lifestyle interventions in high-risk individuals. In summary, although several complex networks of interacting pathways controlling BP have been established (e.g., RAAS and ENaC-related pathways), the current efforts on rare variants analysis have not yet provided a clear answer on where the missing heritability lies.

The advent of NGS provided the opportunity to detect, in a high-throughput way, the entire spectrum of genomic variation ranging from rare to common variants and from SNVs to insertions, deletions, and copy number variants. Despite the undeniable advantages, few studies have been conducted so far using NGS technologies in relation to hypertension and/or BP [61,62,63,64,65,66,67]; (Table 2). WES and, more so, WGS costs are still too high to analyse the large sample size required to identify rare variants. Target re-sequencing allows the cutting of laboratory costs and increases the statistical power by reducing multiple signals testing, therefore, this approach could be useful to detect causative variants underlying the trait by deeply analysing BP-associated loci described by GWAS. However, the studies reported here failed to identify new rare variants, likely because of the reduced sample size compared to GWAS [63,64]. The joint effort of large consortia with available sequencing data would be helpful to meet the need of a larger sample size.

Novel statistical approaches have been developed to overcome the limit imposed by the extremely low frequencies. Also, these tests attempt to take into account the high heterogeneity of the genetic regions in which both common and rare as well as causative and non-causative variants are more likely to occur [99]. However, detecting the few true causative variants among the large number of non-coding variants arising from NGS still represents a big challenge, and additional improvements to better annotate and filter the variants are required.

Another main limitation of rare variants analysis is the study of gene-gene and gene-environment interactions at a population level, which can be investigated only in terms of burden and collapsing tests, with environmental factors playing, anyway, an important role in systemic hypertension. Functional in vitro and in vivo models should further support the statistical interactions.

Rodent models represent an attractive genetic resource to functionally evaluate previously identified rare variants overlapped with human loci. Several rat and mouse strains have been developed for complex phenotypes, including hypertension, and exploited to perform QTL analysis and genome sequencing [100,101,102,103,104]. Here, we reported the study of Vecchione et al. [27], in which, thanks to experimental models, the authors clarified how a rare variant within the *BPIFB4* gene, a possible genetic risk factor for high BP, was implicated in the BP homeostasis by altering eNOS signaling.

It should also be considered that, as hypertension is an age-related condition, additional longitudinal studies incorporating repeated measures of BP would be advantageous. Lastly, most findings should be treated as trait-specific (SBP, DBP, PP, MAP, or hypertension) and population-specific. The majority of studies reported findings deriving from European populations. Allele frequencies and hypertension risk may differ among different geographic regions because of a selective pressure that occurred during the Out-of-Africa Expansion [105].

In conclusion, as sequencing costs will sufficiently decrease to ensure the proper sample size, and novel bioinformatic and biostatistical tools will be available for appropriate analyses, the identification of functional rare and low-frequency variants could really contribute to solving the high complexity of the genetics of hypertension and to elucidate whether new genes might improve patients care and the stratification of patients to distinguish those who will respond best to antihypertensive treatments.

## Figures and Tables

**Table 1:** Results from Exome Chips-Based Studies.

N	Technology	Design	Population	BP Trait	Statistical Analysis	Main Results	References
Discovery: 517 Replication: 57,234	Infinium OmniExpress Exome-Illumina	Linkage analysis in 130 families from CFS to identify rare, coding variants	Whites	SBP, DBP, PP	Family-based burden; SKAT	Linkage peak observed on Chr. 16p13 (MLOD = 2.81) for SBPMultiple rare, coding variants in *RBFOX1* associated with reduced SBP	He et al. [35]
14,028	Illumina ExomeChip	Pleiotropic effects of lipid-associated loci on 10 cardiometabolic traits	Korean	SBP, DBP	GLM	3 SNPs associated with SBP and DBP. Effect sizes (se): −1.5 ± 0.3–−0.78 ± 0.20; *p* < 1.09 × 10^−4^	Kim et al. [38]
~475,000	Affymetrix UK Biobank Axiom Array and Affymetrix UK BiLEVE Axiom Array	Meta-analysis of CHARGE, European-led, and UK Biobank Exome Consortia to identify BP-associated SNVs	~423,000 European	SBP, DBP, PP	EPACTS; EMMA eXpedited; GEMMA	21 SNVs associated with at least 1 BP trait with *p* < 5 × 10^−8^; βs(se): −1.14(0.19)−0.42(0.06)	Kraja et al. [36]
Discovery: 146,562. Follow-up: 180,726. Meta-analysis: 327,288	Illumina ExomeChip	Meta-analysis of CHARGE+, CHD Exome+, ExomeBP, T2D-Genes, GoT2DGenes consortia to identify functional coding variants	All ancestries	SBP, DBP, PP, MAP, HTN	SKAT; Burden test	31 new loci associated with BP (*p* < 3 × 10^−7^; 28 common and 3 low-frequency variants) explaining 0.7% and 1.3% of interindividual variation in SBP and DBP, respectively*PTPMT1*, *DBH*, *NPR1* genes had aggregated rare and low-frequency variants associated with BP (*p* < 9 × 10^−7^)	Liu et al. [33]
15,914	Illumina ExomeChip	Meta-analysis of AADM, ARIC, CARDIA, GenNet, GENOA, HUFS, HyperGEN, LUC cohorts to identify new genes and SNVs across the full frequency spectrum	African ancestry	SBP, DBP	SKAT; T1 burden test; burden-T1-del	9 rare SNVs (mostly missense) within 8 genes (*SLC28A3*, *KRBA1*, *SEL1L3*, *YOD1*, *COL6A1*, *CRYBA2*, *GAPDHS*, and *AFF1*) associated with SBP or DBP (Betas_st_:1.16-3.97; *p* < 5 × 10^−7^)2 significant genes (*CCDC13*, *QSOX1*) for SBP and DBP were also described (Betas_sm_: 54.38 (10.68) and 32.93 (7.13), respectively; *p* < 9.95 × 10^−6^)	Nandakumar et al. [34]
5453	Illumina ExomeChip	Identifying stop-coding variants	Swedish	SBP, DBP	GLM	19 SNVs associated with SBP*PDE11A* R307X mutation: 7 mmHg higher SBP-4.6 mmHg higher DBP	Ohlsson et al. [31]
2045	Illumina ExomeChip	79,578 low-frequency variants analysis within the HyperGEN cohort	African Americans	SBP	CAST; CMC; w-SUM; SST; VT; C-alpha; SKAT; SKAT-O; Minimum P; Fisher’s statistic; RBS; FPCA; Higher criticism	No genome-wide significant results	Sung et al. [28]
Discovery: 192,763 Replication: 155,063	Illumina ExomeChip	Meta-analysis of CHD Exome+, ExomeBP, and GoT2D/T2D-GENES consortia with independent replication within CHARGE + consortium to identify novel coding variants	European: 290,989, South Asian: 27,487, African American, Hispanics and SAS ancestries: 29,350	SBP, DBP, PP, HTN	SKAT; Burden test	Discovery: 51 loci associated with at least one BP traits with *p* < 5 × 10^−8^ Replication: 30 novel SNVs (*p* < 6 × 10^−4^; βs: −1.43−2.70)Rare putative functional variants were identified within *A2ML1*, *COL21A1*, *RRAS*, *RBM47*, and *ENPEP* genes	Surendran et al. [32]
3165	AffymetrixGenome-WideHumanSNP6.0 Array and Illumina ExomeChip	Analysis of common and rare variants in *PCSK9* gene within HyperGEN study and REGARDS population	African-American	SBP, DBP, HTN	SKAT; Joint effect	GWAS: rs12048828 and rs9730100 marginally associated with DBP (βs = 1.8 and 1.0; *p* = 0.05). No significant associations with SBPRare variants: higher median SBP and DBP for carriers of non-synonymous SNPs compared to non-carriers (median SBP and DBP for non-carriers: 127 mmHg and 73 mmHg) Significant cumulative effect of rare variants with DBP (*p* = 0.04) but not with SBP (*p* = 0.14) in HyperGEN Significant cumulative effect of non-synonymous or stop-gain SNPs (*p* = 0.02) but not synonymous SNPs (*p* = 0.73) The joint effect of rs12048828, rs9730100, and 19 rare variants was not statistically significantly associated with DBP (*p* = 0.07) or SBP (*p* = 0.53) The joint effect of the 2 GWAS SNPs and 16 non-synonymous SNPs was significant for DBP (*p* = 0.03) but not for SBP (*p* = 0.41) *PCSK9* rare variants had a cumulative significant association with SBP (*p* = 0.04) but not with DBP (*p* = 0.36) in REGARDS data. Same results when restricted to 15 non-synonymous SNPs (*p* = 0.04 for SBP, *p* = 0.40 for DBP)	Tran et al. [29]
Discovery: 140,886 Replication: >330,000	Customized array with genome-wide imputation based on 1000 Genomes and UK10K sequence data	Analysis of SNVs with MAF ≥ 1% and MAF ≥ 0.01% within UK Biobak	European	SBP, DBP, PP	Linear regression	107 loci validated with *p* < 5 × 10^−8^ The impact of the combination of all loci accounts for 9.3 mmHg higher SBP and over 2-fold higher risk of HTN	Warren et al. [37]
6026	Infinium HumanExome-12 ver. 1.2 BeadChip and Infinium Exome-24 ver. 1.0-Illumina	Longitudinal EWAS for HTN	Japanese	SBP, DBP	GEE model	7 HTN-related SNVs detected, 6 of these variants were located at 12q24.1, creating an East Asian-specific haplotype comprising five derived alleles People carrying the East Asian-specific haplotype displayed a HTN prevalence significantly lower than those individuals carrying a common haplotype. A SNV in *COL6A5* gene was significantly associated with SBP	Yasukochi et al. [30]

Sample number (N), Systolic Blood Pressure (SBP), Diastolic Blood Pressure (DBP), Pulse Pressure (PP), Mean Artery Pressure (MAP), Hypertension (HTN), Single Nucleotide Polymorphism (SNP), Genome-Wide Association Studies (GWAS), Single Nucleotide Variant (SNV), Minor Allele Frequency (MAF), Exome-Wide Association Studies (EWAS), Cleveland Family Study (CFS), Cohorts for Heart and Aging Research in Genomic Epidemiology (CHARGE), Atherosclerosis Risk in Communities (ARIC), Coronary Artery Risk Development in Young Adults (CARDIA), Africa America Diabetes Mellitus (AADM), The Genetic Epidemiology Network of Arteriopathy (GENOA), Howard University Family Study (HUFS), Hypertension Genetic Epidemiology Network (HyperGEN), Loyola University Chicago (LUC), Congenital heart disease (CHD) Exome+, The Genetics of Type 2 Diabetes Consortium (GoT2D)/Type 2 Diabetes Genetic Exploration by Next-generation sequencing in multi-Ethnic Samples (T2D-GENES), REasons for Geographic And Racial Differences in Stroke (REGARDS), Framingham Heart Study (FHS), Standard Error (se), Beta as standardized mean difference (Betas_st_), SeqMeta Beta (Betas_sm_), Efficient and Parallelizable Association Container Toolbox (EPACTS), Efficient Mixed-Model Association eXpedited (EMMA eXpedited), Genome-Wide Efficient Mixed Model Association (GEMMA), Sequence Kernel Association Test (SKAT), Optimal Unified Test (SKAT-O), SKAT-Combined (SKAT-C), Cohort Allelic Sums Test (CAST), Combined Multivariate and Collapsing (CMC), Weighted-Sum (w-SUM), Simple Sum Test (SST), Variable-Threshold (VT), Replication-Based Weighted-Sum Statistic (RBS), Functional Principal Components Analysis (FPCA), Generalized Estimating Equation model (GEE model), burden test on deleterious variants (burden-T1-del).

**Table 2:** Results from Next-Generation Sequencing Studies.

N	Technology	Design	Population	BP Trait	Statistical Analysis	Main Results	References
1851	WES	Haplotype association analysis for *ULK4* and *MAP4* genes within the GAW19 data set	Mexican American	SBP, DBP, HTN	SKAT; SKAT-O; SKAT-C	36 rare haplotype blocks associated with BP in *ULK4* gene and 10 in *MAP4* gene	Datta et al. [61]
1985 unrelated subjects and 1140 relatives	WES	Screening of *SLC12A3*, *SLC12A1* and *KCNJ1* genes exons to identify rare variants within FHS offspring cohort	Largely whites of European descent	SBP, DBP	Two-tailed paired t-test	30 different mutations observed Mean long-term SBP among mutation carriers was 6.3 mmHg lower than the mean of the cohort (*p* = 0.0009). For DBP, mean effect was −3.4 mmHg (*p* = 0.003)	Ji et al. [62]
4178	Target-re-sequencing	Case-cohort study design within the CHARGE Targeted Sequencing Study on 6 BP loci	European	SBP, DBP, PP, MAP	Kernel association test	None of the common variants reached statistical significance threshold of *p* = 0.0001 Rare variation was not significantly associated with any of the BP measures	Morrison et al. [63]
92 (HYPERGENES study)2722 (BP cohort)2013 (HTN cohort)	Target-re-sequencing	Target re-sequencing of a 140-Kb DNA region of Chr. 7 to identify causal or functional variants tagged by the rs3918226 SNP	Flemish	SBP, DBP, HTN	Multivariable-adjusted models	61 novel variants detected by DNA sequencing and confirmed by array-based genotyping rs3918226 remained the SNP most closely associated with HTN The risk allele was associated with lower transcriptional activity of the *eNOS* gene	Salvi et al. [64]
103	WGS	Case-control study on rare variants in unrelated subjects within GAW18 data set	Mexican American	SBP, DBP, HTN	qMSAT; C-alpha; CMC	Rare variants in *SETX* gene intronic region were significantly associated, as aggregate, with hypertension (OR = 9.5, 95% CI (3.43, 28.70); *p* = 8.8 × 10^−7^)	Wang and Wei. [65]
Discovery: 14,497 in first stage and 3459 in second stage	WES	To examine the impact of rare variants in CHARGE and ESP studies with meta-analysis of two-stage discovery cohorts	European and African ancestry	SBP, DBP, PP, MAP	T1; SKAT	95 rare coding variants identified in *CLCN6* associated, in aggregate, with decreased BP (3–4 mmHg), independent of the tagging SNP rs17367504 previously identified The effect size was about four- to six-fold larger than previous common BP variants from GWAS	Yu et al. [66]
142	WGS	Test for the effects of both rare and common variants across the whole genome of unrelated individuals within the GAW18 study	Mexican American	SBP, DBP, HTN	FBAT; GCTA; SKAT	Significant windows within Chr. 3 were reported for associations with SBP and DBP. The most represented gene was *MAP4*	Zhao et al. [67]
1509 unrelated subjects; 256 individuals in 47 families	WGS and WES	To apply CAPL-burden and CAPL-SKAT tests to the GAW19 data set using the combined family and case–control data for HTN (GAW19)	Mexican American	SBP, DBP, HTN	CAPL-burden; CAPL-SKAT	None of the tests for the top 10 genes passed the multiple testing correction threshold (*p* = 3.4 × 10^−6^)	Lin et al. [68]
142	WGS	WGS and gene expression joint analysis in relation to SBP, DBP, and HTN (GAW19)	Mexican American	SBP, DBP, HTN	Weighted U approach	No gene reached statistical significance after adjusting for multiple testing	Tong et al. [69]
1851	WES	To apply W-test on real NGS data set of hypertensive disorder (GAW19)	Mexican American	SBP, DBP, HTN	W-test	*MACROD1/LRP16* locus was associated with HTN after Bonferroni correction (OR = 3.8; *p* = 6.1 × 10^−7^)	Sun et al. [70]
275 trios	WGS	To analyse rare variants within *ADCY5* and *UBE2E2* genes in parent-child trios (GAW18)	Mexican American	SBP, DBP, HTN	Trio-SVM	*ADCY5* and *UBE2E2* genes showed marginal association with HTN with *p* = 3.2 × 10^−4^ for *ADCY5* and *p* = 0.035 for *UBE2E2*	Lu and Cantor. [71]
103 unrelated individuals	WGS	To analyse rare variants from Chr. 3 (GAW18)	Mexican American	SBP, DBP, HTN	SKAT-O	No significant results in the analysis of real phenotype data (*p* = 5.6 × 10^−5^ for coding variants; *p* = 6.9 × 10^−5^ for changing variants; *p* = 1.1 × 10^−4^ for damaging variants)	Derkach et al. [72]
783 (GWAS); 506 (WGS)	WGS	To apply USR algorithm to data from GAW18	Mexican American	SBP, DBP, HTN	USR algorithm	23 promising genes and 3 significant pathways relevant to HTN identified (*p* < 5.28 × 10^−3^)	Cao et al. [73]

Sample number (N), Systolic Blood Pressure (SBP), Diastolic Blood Pressure (DBP), Pulse Pressure (PP), Mean Artery Pressure (MAP), Hypertension (HTN), Single Nucleotide Polymorphism (SNP), Genome-Wide Association Studies (GWAS), Whole Genome Sequencing (WGS), Whole Exome Sequencing (WES), Genetic Analysis Workshop (GAW), Cohorts for Heart and Aging Research in Genomic Epidemiology (CHARGE), Exome Sequencing Project (ESP), Sequence Kernel Association Test (SKAT), Optimal Unified Test (SKAT-O), SKAT-Combined (SKAT-C), Quality-based Multivariate Score Association Test (qMSAT), Combined Multivariate and Collapsing (CMC), Family-based Association Test (FBAT), Genome-wide Complex Trait Analysis (GCTA), Combined Association in the Presence of Linkage (CAPL), support vector machine (SVM), Unified Sparse Regression (USR), Odds Ratio (OR).

## Abbreviations

SBP	Systolic Blood Pressure
DBP	Diastolic Blood Pressure
PP	Pulse Pressure
MAP	Mean Artery Pressure
HTN	Hypertension
SNV	Single Nucleotide Variant
SNP	Single Nucleotide Polymorphism
MAF	Minor Allele Frequency
EWAS	Exome Wide Association Studies
GWAS	Genome-Wide Association Studies
NGS	Next Generation Sequencing
WGS	Whole Genome Sequencing
WES	Whole Exome Sequencing
CFS	Cleveland Family Study
CHARGE	Cohorts for Heart and Aging Research in Genomic Epidemiology
ARIC	Atherosclerosis Risk in Communities
CARDIA	Coronary Artery Risk Development in Young Adults
AADM	Africa America Diabetes Mellitus
GENOA	The Genetic Epidemiology Network of Arteriopathy
HUFS	Howard University Family Study
HyperGEN	Hypertension Genetic Epidemiology Network
CHD	Congenital heart disease Exome+
T2D-GENES	The Genetics of Type 2 Diabetes Consortium (GoT2D)/Type 2 Diabetes Genetic Exploration by Next-generation sequencing in multi-Ethnic Samples
REGARDS	REasons for Geographic And Racial Differences in Stroke
FHS	Framingham Heart Study
GAW	Genetic Analysis Workshop
ESP	Exome Sequencing Project
Beta_sst_	Beta as standardized mean difference
Beta_ssm_	SeqMeta Beta
EPACTS	Efficient and Parallelizable Association Container Toolbox
EMMA eXpedited	Efficient Mixed-Model Association eXpedited
GEMMA	Genome-Wide Efficient Mixed Model Association
SKAT	Sequence Kernel Association Test
SKAT-O	Optimal Unified Test
SKAT-C	SKAT-Combined
CAST	Cohort Allelic Sums Test
CMC	Combined Multivariate and Collapsing
w-SUM	Weighted-Sum
SST	Simple Sum Test
VT	Variable-Threshold
RBS	Replication-Based Weighted-Sum Statistic
FPCA	Functional Principal Components Analysis
FBAT	Family-based Association Test
GEE model	Generalized Estimating Equation model
burden-T1-del	burden test on deleterious variants
qMSAT	Quality-based Multivariate Score Association Test
GCTA	Genome-wide Complex Trait Analysis
CAPL	Combined Association in the Presence of Linkage
SVM	support vector machine
USR	Unified Sparse Regression
GLM	Generalized Linear Model
OR	Odds Ratio
N	Sample number
se	Standard Error
CI	Confidence Interval
